# Protective Potentials of Alpha-Lipoic Acid against Ionizing Radiation-Induced Brain Damage in Rats

**DOI:** 10.1155/2023/4999306

**Published:** 2023-02-03

**Authors:** Ji Xu, Ameer A. Alameri, Rahman S. Zabibah, Gamal A. Gabr, Andrés Alexis Ramírez-Coronel, Hamed Bagheri, Razzagh Abedi-Firouzjah

**Affiliations:** ^1^Department of Rehabilitation Medicine, 3201 Hospital, Hanzhong 723000, China; ^2^University of Babylon, Babylon, Iraq; ^3^Medical Laboratory Technology Department, College of Medical Technology, The Islamic University, Najaf, Iraq; ^4^Department of Pharmacology and Toxicology, College of Pharmacy, Prince Sattam Bin Abdulaziz University, Al-Kharj 11942, Saudi Arabia; ^5^Agricultural Genetic Engineering Research Institute (AGERI), Agricultural Research Center, Giza, Egypt; ^6^Azogues Campus Nursing Career, Health and Behavior Research Group (HBR), Psychometry and Ethology Laboratory, Catholic University of Cuenca, Ecuador; ^7^Doctorate in Psychology, University of Palermo, Buenos Aires, Argentina; ^8^Epidemiology and Biostatistics Research Group, CES University, Colombia; ^9^Radiation Sciences Research Center (RSRC), AJA University of Medical Sciences, Tehran, Iran; ^10^Radiation Biology Research Center, Iran University of Medical Sciences, Tehran, Iran; ^11^Department of Medical Physics Radiobiology and Radiation Protection, School of Medicine, Babol University of Medical Sciences, Babol, Iran

## Abstract

**Background:**

This study was aimed at determining the effects of alpha-lipoic acid on ionizing irradiation-induced oxidative damage and apoptosis in the brain of rats.

**Methods:**

The animals were exposed to whole-brain X-radiation with a 15 Gy single dose in the absence or presence of alpha-lipoic acid (200 mg/kg body weight) pretreatment for one week. The rats were divided into four groups (5 rats in each group): vehicle control, alpha-lipoic acid alone (ALA), radiation alone (RAD), and radiation plus alpha-lipoic acid (RAD+ALA). In the next stage, malondialdehyde (MDA), nitric oxide, catalase (CAT), superoxide dismutase (SOD), and glutathione peroxidase (GPx) in the brain tissue of the rats were measured. Furthermore, the Western blot analysis technique was performed to assess the NOX2, NOX4, and caspase-3 protein expression levels.

**Results:**

Twenty-four hours after the irradiation, MDA and nitric oxide levels in the irradiated rats were significantly higher than those in the control group (*p* < 0.001); however, the pretreatment with alpha-lipoic acid resulted in a significant reduction in these stress oxidative markers (*p* < 0.05). Moreover, a significant decrease in CAT, SOD, and GPx levels was observed in the radiation group alone compared to the control group (*p* < 0.01); in contrast, the activities of these antioxidant enzymes significantly increased in the radiation plus alpha-lipoic acid group in comparison to the radiation group alone (*p* < 0.05). The results of Western blot analysis revealed that NOX2, NOX4, and caspase-3 protein expressions significantly elevated in the irradiated rats compared to the control group (*p* < 0.001). The pretreatment with alpha-lipoic acid could significantly decrease the expression levels of NOX2, NOX4, and caspase-3 in comparison with the radiation group alone (*p* < 0.05).

**Conclusion:**

According to the obtained findings, it can be mentioned that the alpha-lipoic acid pretreatment could mitigate the ionizing irradiation-induced oxidative damage and apoptosis in the brain of the rats.

## 1. Introduction

Brain and other central nervous system (CNS) cancers are among the most fatal malignant tumors [1]. The standard modalities for treating these tumors are surgery, radiation therapy, and chemotherapy [2–4].

Although radiation therapy is an effective therapeutic modality for treating various malignant tumors, the normal cells/tissues around the treatment volume are inevitably irradiated which results in serious adverse effects [5, 6]. Due to the close proximity of normal brain parenchyma and critical structures, CNS radiation therapy or whole brain irradiation is associated with different side effects including neurodegeneration and cognitive disorders [7–11]. Therefore, special attention should be considered for minimizing the ionizing radiation-induced damage to healthy cells/tissues outside the tumor.

Some studies have reported a number of various strategies to improve the radiotherapeutic efficacy or ameliorate the ionizing radiation-induced toxicity, including (1) the combination of radiation therapy with other cancer treatment modalities (e.g., surgery or chemotherapy), which the aim of the combined therapy is the enhancement of tumor cell sensitivity to radiation therapy [12, 13]; (2) the use of advanced radiation therapy techniques (e.g., stereotactic radiosurgery, stereotactic radiotherapy, intensity-modulated radiation therapy, or image-guided radiation therapy) which can result in a more radiation dose to the tumor without an increase in the radiation dose to the surrounding normal organs [13]; and (3) the use of herbal and natural products as radioprotective and radiosensitizing agents for reducing the radiation therapy-induced toxicity [14–22].

The tendency to use the herbal and natural products in order to increase the tumor cell sensitivity to the ionizing radiation and/or mitigate the adverse effects induced by radiation therapy has attracted much attention during the past years [4, 23]. Alpha-lipoic acid is a natural compound and is found in a variety of plant and animal sources, including spinach, tomato, broccoli, kidney, and liver [24]. It is able to quench many free radical species, chelate redox active transitional metals, and prevent membrane lipid peroxidation (LPO) and protein damage through interactions with vitamin C and glutathione (GSH) [25, 26]. Besides, alpha-lipoic acid is able to attenuate the renin-angiotensin system and inhibit nonenzymatic glycation [25, 27]. Moreover, the anti-inflammatory, antiapoptotic, and antioxidant activities of alpha-lipoic acid have been reported in some studies [23, 28, 29]. It is also shown that the use of alpha-lipoic acid can be effective in the improvement of different pathological processes such as Alzheimer [30], human immunodeficiency virus activation [31], hypertension [32], diabetes [33], and ischemia-reperfusion injury [34].

The brain is vulnerable to oxidative damage because of its high oxygen consumption and relatively low levels of antioxidants [35]. Moreover, the high concentration of unsaturated fatty acids in the brain makes this tissue a proper substrate for the occurrence of peroxidation reactions [22, 36]. Therefore, supplementation of antioxidant agents might mitigate the adverse effects of the ionizing radiation on the brain. The importance of the above-mentioned subject and the minimal relevant data in the literature were the main motivations for the current study to investigate the radioprotective potential of alpha-lipoic acid on the ionizing irradiation-induced oxidative damage and apoptosis in the brain of rats.

## 2. Materials and Methods

### 2.1. Animals

The current experimental study was performed on adult male Wistar rats weighing 180-200 g. The animals were kept in standard cages at 5 rats/cage for one week under a condition of 12 hr lightness/12 hr darkness at 22–24°C temperature with 50–55% humidity before conducting the experiment. The rats had free access to food and water. Moreover, all the experimental procedures on the animals were conducted in accordance with the ethical guidelines of AJA University of Medical Sciences (ethical code: IR.AJAUMS.REC.1399.237).

### 2.2. Drug

Alpha-lipoic acid was first dissolved in 10% dimethyl sulfoxide and then diluted into saline at a concentration of 6 mg/ml. The dose of alpha-lipoic acid was selected at 200 mg/kg body weight which was a safe dose based on the previous experimental studies [37–39].

### 2.3. Study Design and Irradiation

After the acclimatization, 20 rats were divided into 4 groups of 5 animals: Group 1 (vehicle control): the rats were orally administrated with saline for 7 days; Group 2 (alpha-lipoic acid alone): the rats were orally pretreated with 200 mg/kg body weight/day of alpha-lipoic acid for 7 consecutive days; Group 3 (radiation alone): the rats were exposed to a 15 Gy single-dose whole-brain irradiation; and Group 4 (radiation plus alpha-lipoic acid): the rats were pretreated orally as drinking water at 200 mg/kg body weight/day of alpha-lipoic acid for 7 consecutive days, followed by a 15 Gy single-dose whole-brain irradiation. Of note, the experimental interval (7 days) was selected based on the previous study [40].

It is noteworthy that 30 min before the irradiation, the rats were anesthetized with an intraperitoneal injection of 60 mg/kg ketamine and 20 mg/kg xylazine. Afterward, the animals were irradiated by 6 MV X-rays generated by a Siemens Primus linear accelerator (Siemens AG, Erlangen, Germany) at a source to axis distance of 100 cm, field size of 10 × 40 cm^2^, dose rate of 200 cGy/min, and gantry angle of 0°.

### 2.4. Preparation of Tissue Sample and Biochemical Analysis

Twenty-four hours after the irradiation process, the rats were anesthetized by intraperitoneal injection of 60 mg/kg ketamine and 20 mg/kg xylazine. The brain of each rat was immediately harvested and washed with phosphate-buffered saline. Each brain was then transferred to a test tube and stored at a -80°C temperature until the samples were applied for preparation of homogenates. For the following measurements, each stored brain tissue was finely minced and homogenized in 100 mM phosphate buffer (pH 7.4), and the tubes were then centrifuged at 300 × g for 2 min at room temperature.

#### 2.4.1. Measurement of Malondialdehyde

Malondialdehyde (MDA) levels were measured based on the method presented by Ohkawa et al. [41] to determine LPO in the brain tissue of the rats in terms of pmol/mg protein [42].

#### 2.4.2. Measurement of Nitric Oxide

Nitric oxide levels were determined in the brain tissue of the rats in terms of *μ*mol/mg protein by measuring the brain supernatant metabolites using the Griess reagent, based on the method presented by Moshage et al. [43].

#### 2.4.3. Measurement of Antioxidant Markers

The activities of catalase (CAT), superoxide dismutase (SOD), and glutathione peroxidase (GPx) were measured using ELISA in accordance with the instructions of the manufacturer (ZellBio GmbH, Germany).

### 2.5. Western Blot Technique

The frozen samples of brain tissues were lysed mechanically in radioimmunoprecipitation assay (RIPA) buffer. The lysates were then centrifuged at 14,000 g for 20 min at 4°C. A 30 *μ*l cell lysate for each sample was separated using 10% sodium dodecyl sulfate polyacrylamide gel electrophoresis. In the next stage, the samples were transferred to polyvinylidene fluoride (PVDF) membranes (Cyto Matin Gene Company, Iran). Afterward, the membrane incubation was done at 4°C overnight in the presence of rabbit anti-NOX2 (Abnova, USA), anti-NOX4 (Novus Biologicals Company, Colorado, USA), anti-caspase-3 (Abcam Biotechnology, Cambridge, UK), and *β*-actin antibodies (Santa Cruz, California, USA). The membranes were washed 3 times for 5 min and then incubated in the appropriate horseradish peroxidase- (HRP-) conjugated secondary antibodies (BioLegend, California, USA) for 1 h at room temperature. Finally, the protein bands were determined using enhanced chemiluminescence and the ECL detection system (Bio-Rad, Philadelphia, PA). The relative densities of NOX2, NOX4, and caspase-3 proteins were determined by ImageJ 1.44 software and normalized to the *β*-actin expression (reported as relative fold changes compared to control).

### 2.6. Statistical Analysis

All data were expressed as mean ± SEM, and the Kolmogorov-Smirnov test was applied to assess the distribution normality of these data. Moreover, we also analyzed the data using one-way analysis of variance (ANOVA) test, followed by Tukey's post hoc test. A *p* ≤ 0.05 was also considered as the level of statistical significance.

## 3. Results

### 3.1. The Effect of Alpha-Lipoic Acid on Oxidative Stress Biomarkers

**Figure 1:**
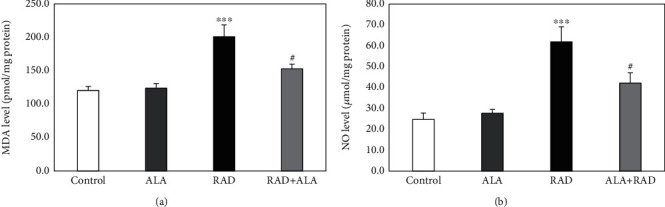
The malondialdehyde (MDA) levels (a) and nitric oxide (NO) levels (b) in the brain tissue of rats in the control, alpha-lipoic acid alone (ALA), radiation alone (RAD), and radiation plus alpha-lipoic acid (RAD+ALA) groups (*n* = 5). Data are presented as mean ± SEM. A significant difference compared to the control (^∗∗∗^*p* < 0.001) and RAD (^#^*p* < 0.05) groups.

Twenty-four hours after the irradiation, the MDA and nitric oxide levels in the brain tissue of the rats were evaluated and the obtained results are shown in Figure 1. According to the findings (Figure 1(a)), a significant increase in the MDA levels of the ionizing radiation-received rats (200.97 ± 17.70 pmol/mg) compared to the control group (120.68 ± 5.90 pmol/mg) was observed (*p* < 0.001). However, a pretreatment with alpha-lipoic acid could significantly reduce the increased levels of MDA in the irradiated rats (152.96 ± 6.91 pmol/mg, *p* < 0.05). Moreover, there were no significant differences in the MDA levels between the alpha-lipoic acid group alone (124.11 ± 6.72 pmol/mg) and the control group (*p* > 0.05).

Figure 1(b) shows the effect of the alpha-lipoic acid pretreatment on the nitric oxide levels in all the experimental groups. It was found that the nitric oxide levels in the brain tissue of the ionizing radiation-treated group were higher than those of the untreated rats (61.92 ± 7.18 vs. 24.78 ± 2.96 *μ*mol/mg, *p* < 0.001). In contrast, the pretreatment with alpha-lipoic acid significantly decreased the nitric oxide levels of the tissue samples (42.10 ± 5.00 *μ*mol/mg) compared to the radiation group alone (*p* < 0.05). No significant differences in the nitric oxide levels between the alpha-lipoic acid group alone (27.67 ± 1.87 *μ*mol/mg) and the control group were observed (*p* > 0.05).

### 3.2. The Effect of Alpha-Lipoic Acid on Antioxidant Biomarkers

**Figure 2:**
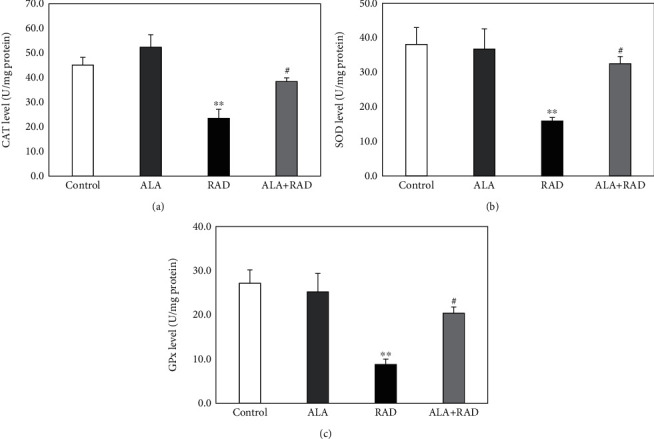
The catalase (CAT) levels (a), superoxide dismutase (SOD) levels (b), and glutathione peroxidase (GPx) levels (c) in the brain tissue of rats in the control, alpha-lipoic acid alone (ALA), radiation alone (RAD), and radiation plus alpha-lipoic acid (RAD+ALA) groups (*n* = 5). Data are presented as mean ± SEM. A significant difference compared to the control (^∗∗^*p* < 0.01) and RAD (^#^*p* < 0.05) groups.

Figure 2 illustrates the effect of alpha-lipoic acid on the CAT, SOD, and GPx activities among all the groups.  Figure 2(a) shows that there was a significant decrease in the CAT levels of the irradiated rats than of the control group (23.46 ± 3.64 vs. 45.10 ± 3.12 U/mg, *p* < 0.01). It was also observed that the antioxidant activity of CAT was significantly increased in the radiation plus alpha-lipoic acid group (38.40 ± 1.51 U/mg) in comparison to the radiation group alone (*p* < 0.05). Furthermore, there were no significant differences in the CAT levels between the alpha-lipoic acid group alone (52.36 ± 5.02 U/mg) and the control group (*p* > 0.05).

Other results (Figure 2(b)) revealed that the SOD levels in the brain tissue of the ionizing radiation-received rats were significantly lower than those of the control group (15.92 ± 1.04 vs. 38.16 ± 4.84 U/mg, *p* < 0.01). Nevertheless, the reduced levels of SOD in the radiation group alone were significantly restored by the pretreatment with alpha-lipoic acid (32.55 ± 2.05 U/mg, *p* < 0.05). It was also found that there were no significant differences in the SOD levels between the alpha-lipoic acid group alone (36.80 ± 5.82 U/mg) and the control group (*p* > 0.05).

In Figure 2(c), it is shown that the GPx levels in the radiation group alone were significantly lower than those in the untreated rats (8.80 ± 1.22 vs. 23.47 ± 2.62 U/mg, *p* < 0.01). In contrast, the pretreatment with alpha-lipoic acid significantly increased the GPx levels of the irradiated rats (20.41 ± 1.40 U/mg, *p* < 0.05). No significant differences in the GPx levels between the alpha-lipoic acid group alone (25.25 ± 4.18 U/mg) and the control group were observed (*p* > 0.05).

### 3.3. The Effect of Alpha-Lipoic Acid on NOX2 and NOX4 Expression Levels

**Figure 3:**
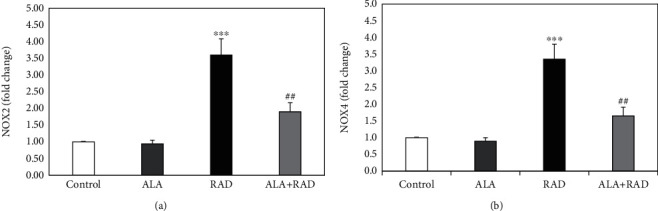
The results of Western blot analysis for the NOX2 protein expression (a) and the NOX4 protein expression (b) in the brain tissue of rats in the control, alpha-lipoic acid alone (ALA), radiation alone (RAD), and radiation plus alpha-lipoic acid (RAD+ALA) groups (*n* = 5). Data are presented as mean ± SEM. A significant difference compared to the control (^∗∗∗^*p* < 0.001) and RAD (^##^*p* < 0.01) groups.

The analyses with Western blot (Figure 3(a)) revealed a 3.60 ± 0.48-fold increase in the NOX2 expression levels of the rats following radiotherapy compared to the unirradiated rats (*p* < 0.001). The pretreatment with alpha-lipoic acid reduced the increased NOX2 expression levels of the irradiated rats to 1.90 ± 0.27-fold (*p* < 0.01).

Figure 3(b) also shows a 3.35 ± 0.45-fold increase in the NOX4 expression levels of the irradiated rats as compared to the control group (*p* < 0.001). However, there was a significant decrease in the NOX4 expression levels of the rats receiving radiation plus alpha-lipoic acid in comparison with the radiation group alone (1.65 ± 0.26-fold, *p* < 0.01).

### 3.4. The Effect of Alpha-Lipoic Acid on Caspase-3 Expression Level

**Figure 4:**
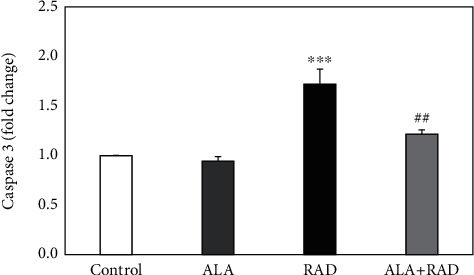
The results of Western blot analysis for the caspase-3 protein expression in the brain tissue of rats in the control, alpha-lipoic acid alone (ALA), radiation alone (RAD), and radiation plus alpha-lipoic acid (RAD+ALA) groups (*n* = 5). Data are presented as mean ± SEM. A significant difference compared to the control (^∗∗∗^*p* < 0.001) and RAD (^##^*p* < 0.01) groups.

The expression levels of caspase-3 protein in all the groups were determined by Western blot analysis, and these results are illustrated in Figure 4. A significant increment in the caspase-3 expression levels of the rats receiving radiation alone was observed compared to the control group (1.72 ± 0.15-fold, *p* < 0.001). In contrast, the pretreatment with alpha-lipoic acid could significantly reduce the caspase-3 expression levels of the irradiated rats compared to those receiving radiation alone (1.22 ± 0.04-fold, *p* < 0.01).

## 4. Discussion

In this experiment, we evaluated the possible protective effects of alpha-lipoic acid on radiation-induced brain injury. The key mechanism of radiation toxicity in normal cells is the induction of oxidative stress, which can lead to cell death. We selected some well-known parameters of oxidative stress to evaluate the effect of alpha-lipoic acid alone and in combination with radiation. MDA, nitric oxide, CAT, SOD, and GPx were considered as the markers of oxidative stress in this study. The administration of alpha-lipoic acid alone did not cause any oxidative stress in brain cells. No significant changes in the levels of these markers in the brain were observed after treatment with alpha-lipoic acid. The irradiation of rat brains led to a significant increase in the levels of MDA and nitric oxide. Furthermore, a significant reduction in the activity of antioxidant defense enzymes including SOD, CAT, and GPx was observed. Our findings also showed that the pretreatment with alpha-lipoic acid results in a reduction in the stress oxidative markers including MDA and nitric oxide and an increase in the antioxidant defense enzymes including SOD, CAT, and GPx of the irradiated rat brains. To date, some studies have reported that alpha-lipoic acid cotreatment can boost the antioxidant defense and reduce the MDA and nitric oxide levels in some irradiated tissues. A study by Manda et al. showed that the pretreatment with alpha-lipoic acid can reverse the decreased total antioxidant capacity in plasma following whole body irradiation of the rats. Moreover, the whole body irradiation suppressed the level of sulfhydryl (natural antioxidant in cells), while treatment with alpha-lipoic acid could ameliorate this reduction in different tissues such as the brain, testis, spleen, kidney, and liver [44]. Another study showed that alpha-lipoic acid reduces the levels of MDA and 8-hydroxy-2′-deoxyguanosine (8-OHdG) in thyroid tissue of the rats following irradiation. This was associated with a remarkable reduction in the release of proinflammatory and profibrosis cytokines [45]. Alpha-lipoic acid has been also shown to ameliorate radiation-induced oxidative stress in fibroblasts by inhibiting the production of ROS and nitric oxide [46]. Similarly, it was shown that alpha-lipoic acid treatment could stimulate the activity of CAT and glutathione S-transferase and reduce MDA in the testis after irradiation [47].

In this study, we also investigated the modulatory effect of alpha-lipoic acid on the expression of NOX2, NOX4, and caspase-3. The results of our experiment showed a significant increase in the expression of NOX2 and NOX4 in the brain tissue of the rats following exposure to the ionizing radiation. The irradiation of rat brains led to a more than 3-fold increase in the protein levels of NOX2 and NOX4. However, alpha-lipoic acid cotreatment could attenuate the expression of these enzymes. These findings indicate that alpha-lipoic acid can decrease ROS generation by these enzymes, thus reducing chronic oxidative stress after exposing the brain tissue to the ionizing radiation, as these results can explain a protective mechanism of alpha-lipoic acid in the brain tissue of the rats. The involvement of NADPH oxidase enzymes in the radiation-induced brain injury has already been investigated in an experimental study. Moreover, exposing the brain tissue of the rats to the ionizing radiation showed a significant increase in the expression of NOX4, p22^phox^, and p47^phox^ in the microvascular endothelial cells. The results also showed an increase in the expression of inflammatory mediators such as NF-*κ*B. However, the genetic inhibition of NADPH oxidase enzymes led to a remarkable reduction in the production of ROS [48]. To date, there was no study evaluating the protection of the brain tissue against the ionizing radiation through the modulation of these enzymes. However, a study confirmed that suppression of NOX4 by alpha-lipoic acid reduces oxidative stress and apoptosis in the bone marrow following the whole body irradiation [49]. Increased expression of caspase-3 after the irradiation shows an increase in the incidence of apoptosis in brain cells. A reduction in the expression of caspase-3 also confirms that alpha-lipoic acid cotreatment protects the brain tissue of the rats against oxidative stress and apoptosis after the irradiation. Our obtained results are supported by the previous studies that have reported the use of alpha-lipoic acid could decrease caspase-3 expression level [50–53].

Experimental studies in the recent decade have indicated a key role of reduction/oxidation (redox) interactions in chronic oxidative stress, inflammation, and normal tissue injury [54]. The main redox system mediators are NADPH oxidases, cyclooxygenase-2, inducible nitric oxide synthase, mitochondria, and antioxidant defense enzymes [55–58]. Overproduction of ROS by mitochondria and prooxidant enzymes like NOX2 and NOX4 can suppress the activity of antioxidant defense enzymes, leading to cell death and tissue injury [59]. NOX2 and NOX4 are among the most important NADPH oxidase subfamilies. It has been reported that the activation and upregulation of these enzymes are associated with oxidative stress, DNA damage, genomic instability, and cell death for a long time after exposure to the ionizing radiation [55, 58]. Furthermore, there is a growing evidence that confirms the pivotal role of these enzymes in neurodegenerative diseases [60]. NOX2 and NOX4 can be activated by endothelial cells, leading to vascular injury [61]. In addition to endothelial cells, experimental studies including both in vitro and in vivo experiments showed that other cells such as fibroblasts, macrophages, neutrophils, and lymphocytes can generate ROS through NADPH oxidases, including NOX2 and NOX4 [55, 62]. Damage to vessels and microvessels in the brain can induce hypoxia and necrosis in normal tissue of the brain. The release of proinflammatory and profibrosis cytokines can increase the expression and activity of NOX2 and NOX4, leading to ROS production and oxidative stress [63]. The administration of some adjuvants to enhance antioxidant defense capacity is an intriguing approach for protecting normal tissues against the ionizing radiation [64]. However, some experiments have suggested the inhibition of prooxidant enzymes as a strategy for protecting normal cells/tissues [14, 65, 66]. In total, the results of this experiment indicate that the inhibition of NOX2 and NOX4 by alpha-lipoic acid is an interesting approach to protect the brain tissue against the toxic effects of the ionizing radiation. In contrast, alpha-lipoic acid cotreatment could reduce oxidative stress and apoptosis in brain cells by boosting antioxidant defense enzymes and reduction of oxidative stress.

## 5. Conclusion

In the present study, the neuroprotective potential of alpha-lipoic acid against the brain damage induced by radiotherapy in the rats was assessed. The results showed that the alpha-lipoic acid pretreatment could mitigate the ionizing radiation-induced oxidative damage and apoptosis in the brain tissue of the rats. However, the use of alpha-lipoic acid (as an adjuvant agent) for protecting the neurotoxicity induced by radiotherapy in cancer patients requires further clinical studies.

## Data Availability

The data used to support the findings of this study are available from the corresponding authors upon request.
